# Development, validation and use of artificial-intelligence-related technologies to assess basic motor skills in children: a scoping review

**DOI:** 10.12688/f1000research.138616.3

**Published:** 2026-04-24

**Authors:** Joel Figueroa-Quiñones, Juan Ipanaque-Neyra, Heber Gómez Hurtado, Oscar Bazo-Alvarez, Juan Carlos Bazo-Alvarez

**Affiliations:** 1 Universidad Autonoma de Ica, Ica, Peru; 2 Instituto de Investigación, Capacitación y Desarrollo Psicosocial y Educativo (PSYCOPERU), Lima, Peru; 3 Ingeniería de Sistemas e Informática, Universidad Tecnológica del Perú, Lima, Peru; 4 School of Medicine, Universidad San Juan Bautista, Lima, Peru; 5 Research Department of Primary Care and Population Health, University College London, London, UK; 6 MedFam Group, School of Medicine, Universidad Cesar Vallejo, Trujillo, Peru

**Keywords:** Basic motor skills, fundamental movements, machine learning, motion detection, prediction techniques

## Abstract

**Background:**

In basic motor skills evaluation, two observers can eventually mark the same child’s performance differently. When systematic, this brings serious noise to the assessment. New motion sensing and tracking technologies offer more precise measures of these children’s capabilities. We aimed to review current development, validation and use of artificial intelligence-related technologies that assess basic motor skills in children aged 3 to 6 years old.

**Methods:**

We performed a scoping review in Medline, EBSCO, IEEE and Web of Science databases. PRISMA Extension recommendations for scoping reviews were applied for the full review, whereas the COSMIN criteria for diagnostic instruments helped to evaluate the validation of the artificial intelligence (AI)-related measurements.

**Results:**

We found 672 studies, from which 12 were finally selected, 7 related to development and validation and 5 related to use. From the 7 technology development studies, we examined their citation networks using Google Scholar and identified 10 subsequent peer-reviewed publications that either enhanced the original technologies or applied them in new research contexts. Studies on AI-related technologies have prioritized development and technological features. The validation of these algorithms was based on engineering standards, focusing on their accuracy and technical performance, but without integrating medical and psychological knowledge about children’s motor development. They also did not consider the technical characteristics that are typically assessed in psychometric instruments designed to assess motor skills in children (e.g., the Consensus-based Standards for the Selection of Health Measurement Instruments “COSMIN”). Therefore, the use of these AI-related technologies in scientific research is still limited.

**Conclusion:**

Clinical measurement standards have not been integrated into the development of AI-related technologies for measuring basic motor skills in children. This compromises the validity, reliability and practical utility of these tools, so future improvement in this type of research is needed.

## Introduction

The development of basic motor skills (BMS) in children aged 3 to 6 years is critical, as this is a period of rapid motor growth, where children acquire physical skills that allow them to participate in a variety of activities. ^1^^,^^2^ At this age, children experience significant improvements in gross motor control, allowing them to perform movements such as running, jumping, and manipulating objects with greater precision. ^3^^,^^4^ The acquisition of these motor skills is essential for physical, cognitive and emotional development, as BMS are strongly linked to general well-being, self-esteem and social integration. ^5^ Early BMS competence may also have long-term biomechanical relevance, as it supports more efficient movement patterns, later participation in physical activity, and potentially lower injury risk across development. ^49^ For example, children with BMS stimulation tend to participate more in physical activities (e.g., school games and sports), suggesting socioemotional and health benefits such as early prevention of obesity. ^6^ Likewise, some studies have designed, implemented, and recommended early interventions to promote healthy BMS development in preschool children. ^7^ To evaluate the efficacy of these interventions and to monitor the optimal development of BMS in children, valid and reliable measurement tools are needed. Typically, BMS assessment relies on trained professionals who observe, record, and score children’s performance on specific motor tasks. ^8^^,^^9^ However, a major challenge in this approach is observer bias. Even when raters receive standardized training, small differences in scoring can introduce variability in BMS measurements. This variability reduces the accuracy of the assessment and can lead to misinterpretations. For example, two children with similar motor skills may receive different scores depending on the assessor, resulting in inconsistent results. When these inconsistencies follow a systematic pattern, they contribute to observer bias, a well-documented source of measurement error. ^10^^,^^11^ Earlier reviews in behavioral research documented substantial shortcomings in the reporting of interobserver reliability and procedures to minimize observer bias. ^12^ Similarly, another review on child development found that the quality of reporting on the use of assessors in these studies was poor and that variability in assessor performance may obscure the true developmental status of children, compromising complex and costly clinical decisions. ^13^

**Figure 1.**
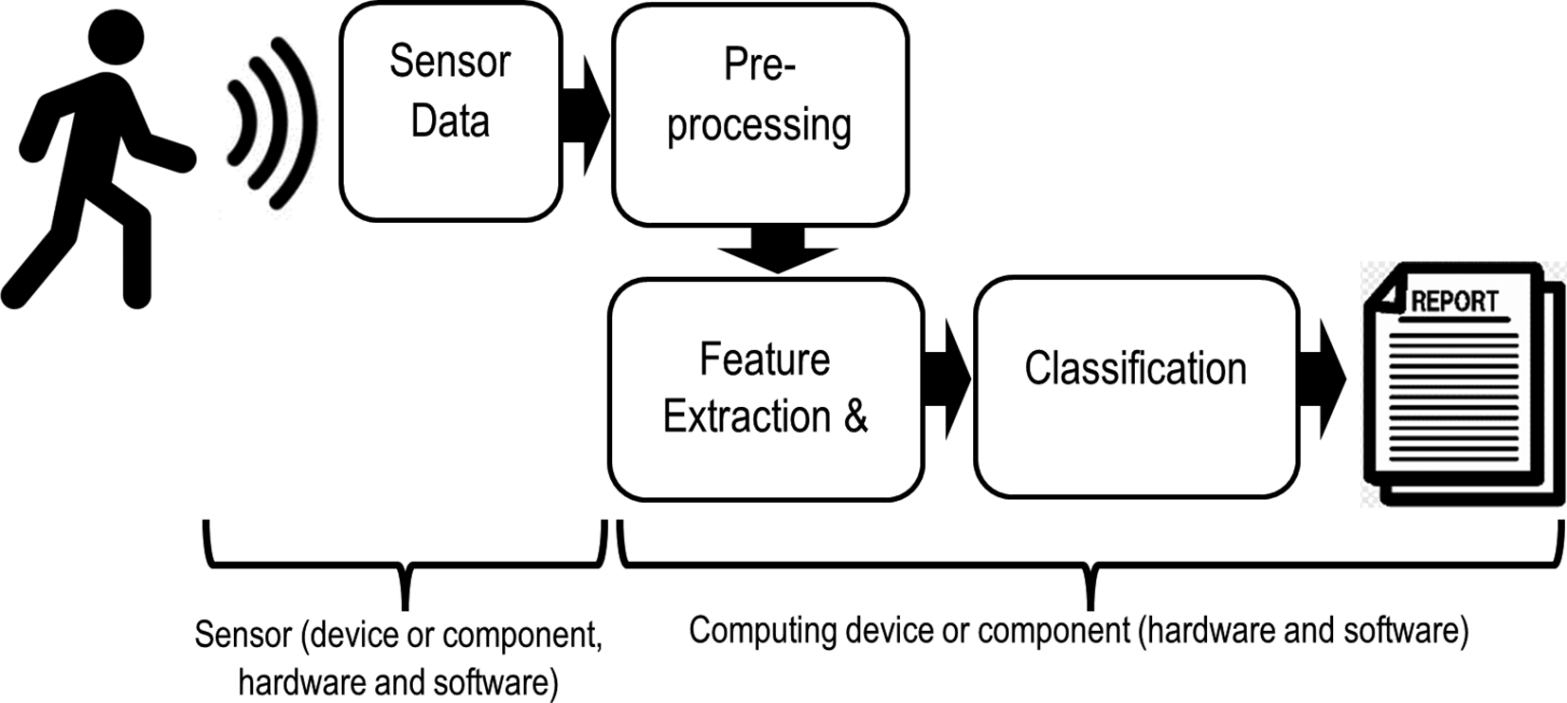
Process of recognition and classification of human motion patterns performed by artificial intelligence (AI)-related technologies.

AI-related technologies (i.e., computational systems that use artificial intelligence to analyze, learn from, and interpret data) offer a promising alternative to minimize observer bias in BMS assessment. ^14^ For example, for motion capture and analysis, computer vision tools such as OpenPose, MediaPipe and DeepLabCut enable pose estimation and tracking of key points of the human body with high accuracy. ^15^ In addition, deep learning techniques, such as Convolutional Neural Networks (CNN) and vision-specialized Transformer Models (ViT), have proven to be effective in classifying motion sequences in videos. ^16^ In that sense, these AI-related technologies for recognizing and classifying human motion patterns consist of several steps ( Figure 1). ^17^ First, sensor or video devices capture data on human movement. Then, these data undergo pre-processing to reduce noise and enhance relevant features. This step often involves filtering techniques, such as Fast Fourier Transformation (which helps separate important movement signals from background noise) or wavelet transforms. Additionally, to simplify complex data and highlight key movement patterns, methods like principal components analysis (which reduces data dimensions while preserving essential information) or linear discriminant analysis (which enhances the distinction between movement categories) are applied. ^17^ Next, feature selection methods come into play, determining a subset of features from the initial set that is highly suitable for subsequent classification while adhering to various optimisation criteria. Among the efficient methods for feature selection are Sequential Forward Selection, which starts with an empty set and iteratively adds the feature that best meets the optimisation criterion, and Backward Selection, which involves removing features from the set in a repetitive manner. However, many contemporary AI pipelines rely on end-to-end deep learning approaches, such as CNNs or vision transformers, which can learn relevant representations directly from images, videos, or pose sequences with limited or no manual feature extraction. Finally, AI or machine learning classifiers are required to identify the corresponding class of motion, in our case, a class that reflects the BMS development of a child ( *e.g.*, delayed, normal or advanced for its age group). Machine learning tools include binary classification trees, decision engines, Bayes classifiers, k-Nearest Neighbour, rule-based approaches, linear discriminant classifiers and Support Vector Machines. More sophisticated deep learning tools, such as neural networks, are also used. From here onwards, we indistinctly use the expression ‘AI-related technology’ for referring to the full process described in Figure 1 or just the classification tools.

The application of AI-related technology in physical performance assessment is rapidly increasing. ^18^ For example, machine learning techniques have been used to assess physical activity intensity in adults. ^19^^,^^20^ A recent review identified at least 53 studies on motion detection using deep learning or machine learning, with 75% of these studies published since 2015. ^21^ AI has also been applied to detect gait abnormalities ^22^ and diagnose health conditions related to walking patterns, ^23^^,^^24^ as well as to identify early motor skill impairments linked to neurodevelopmental disorders. ^25^ Other AI-based algorithms have been implemented to evaluate psychomotor learning performance in students. ^26^^,^^27^ However, despite the increasing use of AI in motor performance assessment, there is no comprehensive review examining its specific application in the assessment of BMS in preschool children, being a crucial stage for early detection and intervention. Moreover, the scope, limitations and validity of AI-based technologies in this context are not yet clearly systematized. Therefore, it is required to synthesize existing knowledge and guide the development of more accurate and accessible assessment tools.

In this study, we aimed to perform a scoping review on studies related to the development and use of AI-related technologies to assess BMS in children. Our objectives were to: 1) determine the general characteristics of the studies; 2) describe the engineering of the AI technologies designed to assess BMS in preschoolers; 3) determine the substantive validation performed on the AI technologies identified, and 4) describe the current use of these AI technologies in applied research.

## Methods

The protocol for this review is available here. ^28^ The PRISMA Extension recommendations for scoping reviews were applied for the full review, whereas the COSMIN guidelines were applied for objective 2. ^29^^,^^30^ The checklists of these guidelines can be found here. ^31^

### Target studies

We were interested in published studies focused on engineering, substantive validation, or the use of AI-related technologies developed to evaluate BMS in children. A study was focused on engineering when it was strictly dedicated to developing algorithms for motor skills recognition and classification. A study was focused on substantive validation when the validity and reliability of the AI-related technology were evaluated following psychometric international standards. ^32^ A study only used AI-related technology when it did not include engineering or validation; in other words, it just used the technology developed by someone else.

We also defined the following criteria for the search: 1) studies in preschool-aged children (3 to 6 years), 2) studies in which the motor ability (motor or play skills) of the child was assessed using AI-related technologies for motion detection, and 3) studies in which at least one of the basic motor skills described in the literature (running, jumping, kicking, throwing, or catching a ball) was measured. In addition, we excluded 1) studies that did not clearly describe the AI-related technology used or developed, 2) opinion articles, editorials, or narrative reviews without empirical data and 3) grey literature (e.g. theses, dissertations, or non-peer-reviewed reports).

### Search strategy

We searched for studies published before January 30, 2023 in the target publications in Medline (SCR_002185), Web of Science (SCR_022706), IEEE (SCR_008314), and EBSCO (SCR_022707). These databases were selected because they specialize in biomedical, engineering, and multidisciplinary research, ensuring that we captured relevant studies in health sciences, AI applications, and motion analysis.

Search terms included keyword combinations such as “child,” “preschool,” “basic motor skills,” “artificial intelligence,” “motion sensing,” and “calibration,” along with related terms and synonyms identified through a preliminary literature review (keywords) and controlled vocabulary (MeSH terms). The full search strategy and complete list of search terms are available here. ^33^

The search formulas were applied to the databases and all the files were exported in RIS format. Then, to ensure an objective selection process, these identified files were uploaded to the Rayyan platform which facilitated blind selection by the reviewers and expedited the identification of duplicates.

The selection process consisted of two phases. In the first phase, titles and abstracts were reviewed by two independent groups (each consisting of two previously trained medical students). To minimize selection bias, the Rayyan blinding function was used, which prevented reviewers from identifying the decisions of the other reviewers until the final selection phase. In addition, allocation of studies to reviewers was randomized within each group to further reduce potential bias. In case of disagreement, a consensus discussion was held among the reviewers. If consensus could not be reached, the principal investigator made the final inclusion decision.

In the second phase, a full-text review was performed following the same procedure, ensuring consistency and methodological rigor. The final set of studies was determined after resolving all discrepancies through consensus discussions and the intervention of the principal investigator.

Additionally, we mapped those studies that updated or used the AI-related technology identified as engineered and validated in the previous step, by exploring the citations/references reported in the latter.

### Data extraction

Data extraction was performed in a structured manner using a pre-designed form. ^34^ To reduce errors and improve the accuracy of the extracted data, one peer reviewer performed the initial extraction and a second peer independently verified the information. Any discrepancies in the extraction were reviewed jointly and/or, with the intervention of the principal investigator. Cross-checks were implemented to ensure the consistency of the information collected. The form included data about the general characteristics of the studies, the engineering of the AI-related technologies, the substantive validation of these technologies, and their current use for BMS assessment in children.

1. General information: First author of the study, country of the study, year of publication, number and sex of participants, health condition ( *e.g.*, children with a medical condition that could influence their motor skills).
2. Engineering: Motion capture interface type, basic composition of technologies, system used for motion capture, type of programming language used for system development or modelling, and technology accessibility.
3. Substantive validation: Type of technology developed and validated, validation method, data collection methods, data for COSMIN (see next section), feasibility and usability of the technology.
4. Use: Type of technology used, training of the evaluation team, reported technology reliability, limitations during the technology use, advantages of the technology application, complementary tools, reference to a publication on the technology used.

### Data analysis

All data collected were summarised as categorical variables, organised and presented in tables, using descriptive statistics such as simple frequencies and percentages. Since this was a scoping review, a narrative synthesis was used to summarize the findings of the studies, focusing on the characteristics and psychometric properties evaluated according to COSMIN standards.

The COSMIN standards were applied to assess the technical quality of the substantive validation of the AI-related technologies for BMS evaluation. ^27^ In practice, these technologies ( *e.g.*, algorithms) work like psychometric tests ( *e.g.*, producing similar BMS measurements); thus, the former can be ‘substantively validated’ as the latter usually are. In this review, COSMIN was applied from a measurement perspective to examine evidence relevant to assessment use, rather than to evaluate algorithmic performance per se. COSMIN is an international standard for reviewing the technical quality of validation studies of psychometric tools ( *e.g.*, tests for measuring BMS).

To perform the COSMIN assessment, two investigators independently assessed and scored eight psychometric properties or indicators (content validity, internal consistency, structural validity, reliability, measurement error, criterion validity, construct validity, and responsiveness). Each indicator was evaluated according to the checklist proposed by Mokkink *et al.* ^35^ For this study, we scored as follows: 1 = N. A, 2 = inadequate, 3 = doubtful, 4 = adequate and 5 = very good. A total score was calculated for each indicator, keeping similar levels for interpretations (very good, adequate, doubtful, inadequate, N.A.). All results from COSMIN assessment were presented in a table.

## Results

**Figure 2.**
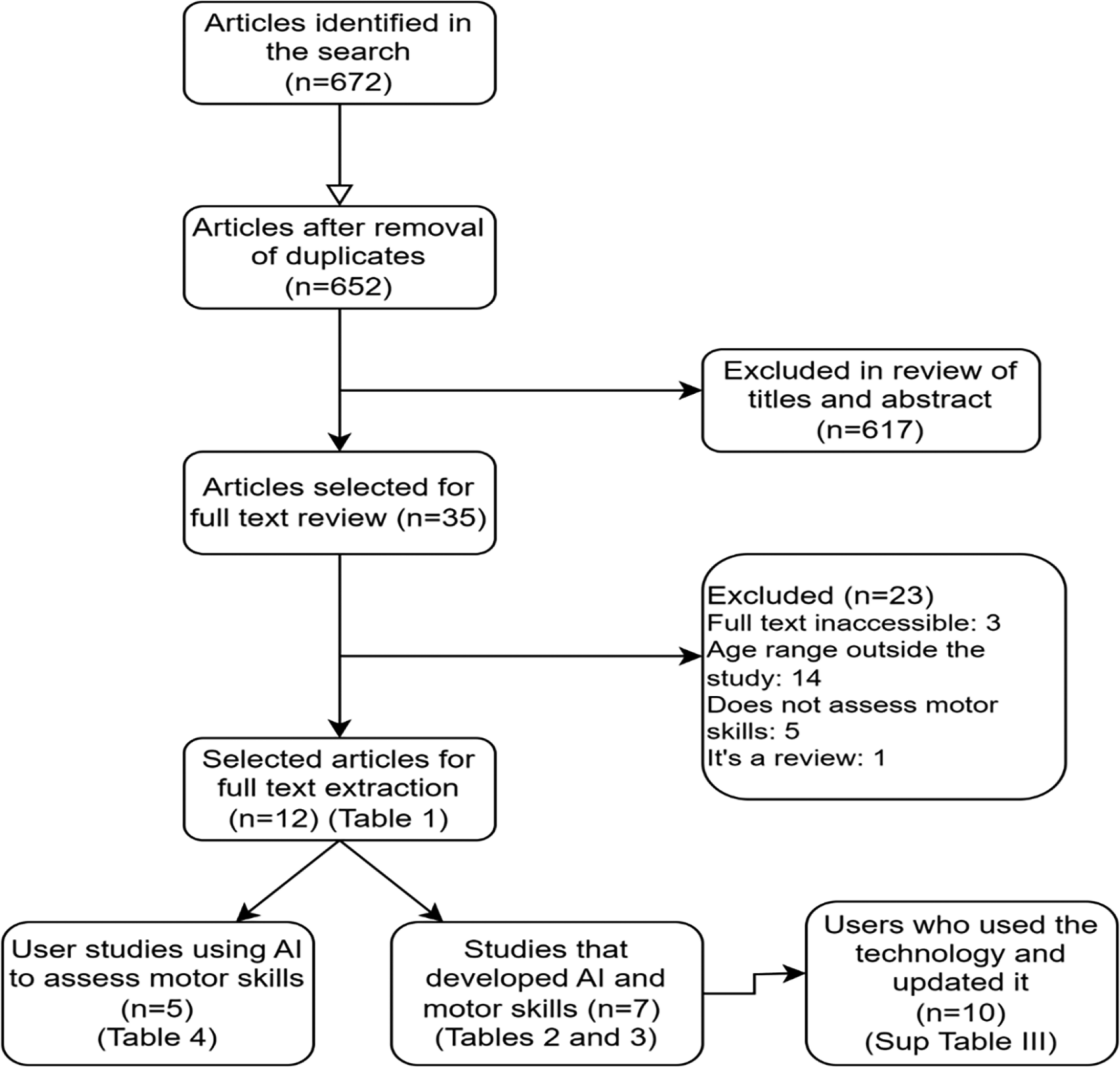
PRISMA diagram for the scoping review.

We identified 672 studies in the first search step, from which 12 studies were finally selected. Among these studies, five were focused on AI-related technology use, while seven were focused on AI-related technology engineering and/or validation ( Figure 2).

During the last decade, most studies were performed in Asian and European countries (n=9/12, 74.9%) ( Table 1). Almost all studies were carried out in children of both sexes (n=9/12, 75%), and only one was focused on children with some type of motor problem.

**Table 1.** General characteristics.

Characteristics of the studies	N=12
Continent	
Asia	5 (41.6)
Europe	4 (33.3)
Latin America	1 (8.33)
North America	1 (8.33)
Oceania	1 (8.33)
Year of publication	
2011-2021	10 (83.3)
≤2010	2 (16.7)
Participant gender	
Boys only	1 (8.33)
Girls only	1 (8.33)
Both boys and girls	9 (75.0)
Not report	1 (8.33)
Population type	
Children without health problems	9 (75.0)
Children with attention and concentration problems	1 (8.3)
Children with some delay in motor development	1 (8.3)
Obese children	1 (8.3)

To capture the child’s movement, researchers mostly used simple devices such as digital video cameras (n=5/7, 71.4%) ( Table 2). More sophisticated devices were less common, such as sensors attached to the body (n=2/7, 28.6%) or multimedia devices connected to personal computers (n=2/7, 28.6%). The software used for each device was different for each study. The most common type of AI-related technology was machine learning tools for movement pattern recognition (n=4/7, 57.1%), while deep learning algorithms were rarely used (n=1/7, 14.3%). Only a few of these tools are free-access (n=2/7, 28.6%). Most codes were implemented in Python (SCR_008394) and supported by libraries such as OpenGL (which produces 2D and 3D graphics) ^36^^–^^38^ and Numpy (SCR_008633) (which creates vectors and matrices, and mathematical functions) (45) that helps to process images that are captured in real-time and obtain an accurate representation of the movement.

**Table 2.** Engineering characteristics of studies that developed artificial intelligence (AI)-related technologies.

Characteristics	N=7
Motion capture device	
Digital cameras	5 (71.4)
Smartphones application	1 (14.3)
iPod touch	1 (14.3)
Other motion capture devices	
Tracker, marker or movement sensor	2(28.6)
Multimedia devices	2(28.6)
Both of them	3 (42.2)
System used for motion capture	
Microsoft Kinect	1 (14.3)
myoMOTION	1 (14.3)
OptiTrack Arena	1 (14.3)
ActiGraph GT3X	1 (14.3)
ProReflex-MCU 240; QualisysMedical AB	1 (14.3)
Acceleration recorder	1 (14.3)
iPod touch (operative system)	1 (14.3)
Type of AI-related tool	
Machine learning for movement patterns recognition	4 (57.1)
Kinematic analysis	2(28.6)
Deep learning and neural networks	1 (14.3)
Accessibility to technology or codes	
Free or open source	2(28.6)
Paid/does not report	5 (71.4)

For the COSMIN evaluation, we considered seven studies that developed a substantive validation of AI technologies ( Table 3). More than half of the studies reported the evaluation of content validity (n=4/7, 57.1%), reliability (n=1/7, 14.2%), and construct validity (n=1/7, 14.2%) with an adequate level. However, other measurement properties, such as structural validity, measurement error and responsiveness, were inadequately or not evaluated in all studies, according to COSMIN standards (n=5/8, 62.5%). These ratings should be interpreted as reflecting limited psychometric validation or reporting, rather than poor algorithmic performance per se, since many included studies primarily focused on engineering development and technical performance. In traditional motor assessment, reliability usually requires evidence of stable scores under similar testing conditions, whereas responsiveness requires evidence that the tool can detect meaningful changes over time or after intervention. Such evidence was rarely reported in the studies included in this review. It was not unusual for studies to declare the evaluation of a psychometric property (e.g., reliability) without reporting the corresponding final results.

**Table 3.** Studies that developed substantive validation of artificial intelligence (AI)-related technology (n = 7) COSMIN Standards.

MEASUREMENT PROPERTY	Study 1	Study 2	Study 3	Study 4	Study 5	Study 6	Study 7
	Shengyan Li (2017)	Santiago Ramos (2014)	Yukie Amemiya (2018)	Hsun-Ying Mao (2014)	Satoshi Suzuki (2019)	Matthew N. Ahmadi (2020)	Parvinpour, S. (2019)
**CONTENT VALIDITY**							
Relevance	INADEQUATE	DOUBTFUL	DOUBTFUL	INADEQUATE	DOUBTFUL	INADEQUATE	DOUBTFUL
Comprehensiveness	DOUBTFUL	ADEQUATE	ADEQUATE	DOUBTFUL	ADEQUATE	DOUBTFUL	ADEQUATE
Comprehensibility	DOUBTFUL	ADEQUATE	ADEQUATE	DOUBTFUL	ADEQUATE	DOUBTFUL	ADEQUATE
**INTERNAL CONSISTENCY**	INADEQUATE	INADEQUATE	INADEQUATE	INADEQUATE	INADEQUATE	INADEQUATE	INADEQUATE
**STRUCTURAL VALIDITY**	INADEQUATE	DOUBTFUL	DOUBTFUL	DOUBTFUL	DOUBTFUL	INADEQUATE	INADEQUATE
**RELIABILITY**	DOUBTFUL	DOUBTFUL	DOUBTFUL	ADEQUATE	DOUBTFUL	DOUBTFUL	DOUBTFUL
				(ICC=0,67)			
**MEASUREMENT ERROR**	INADEQUATE	DOUBTFUL	DOUBTFUL	DOUBTFUL	INADEQUATE	DOUBTFUL	INADEQUATE
**CRITERION VALIDITY**	INADEQUATE	INADEQUATE	INADEQUATE	INADEQUATE	INADEQUATE	INADEQUATE	INADEQUATE
**CONSTRUCT VALIDITY**							
Convergent validity	INADEQUATE	INADEQUATE	ADEQUATE	INADEQUATE	INADEQUATE	INADEQUATE	INADEQUATE
Discriminative validity	INADEQUATE	INADEQUATE	DOUBTFUL	DOUBTFUL	DOUBTFUL	DOUBTFUL	DOUBTFUL
**RESPONSIVENESS**	INADEQUATE	INADEQUATE	INADEQUATE	INADEQUATE	INADEQUATE	INADEQUATE	INADEQUATE

**INADEQUATE:** No adequate analysis, process or method was performed. For example, there was no recording or transcription, there is no mention of how many and who the evaluators were.

**DOUBTFUL:** The analyses, methods and processes carried out are doubtful or unclear.

**ADEQUATE:** Assumable that the methods, processes and analyses were correct, but they are not clearly described.

**VERY GOOD:** An adequate method, procedure and analysis was carried out. Are clearly described within the text.

In studies using AI-related technology, the children’s movements were captured by trained personnel (n=2/5, 40%) using digital cameras or camcorders (n=4/5, 80%) ( Table 4). In addition, some supporting technologies that provide high-quality video motion capture, such as “Quintic Biomechanics software”, was also reported. Users reported some advantages of these technologies; for example, the short-term evaluation needed and precise and consistent measures that allow a detailed analysis of motor skills. However, no formal generalization of the conclusions to larger populations was reported as a technical limitation.

**Table 4.** Current use of studies that used artificial intelligence (AI)-related technology.

Characteristics	N=5
Motion capture device	
Digital camera/camcorder	4 (80.0)
Haptic interface	1 (20.0)
Training for the evaluation team	
Yes	2 (40.0)
No/not reported	3 (60.0)
Reliability of AI-related technology	
Inter- and intra-rater reliability	2 (40.0)
Not reported	3 (60.0)
Limitations reported while using technology	
Yes	1 (20.0)
No/not reported	4 (80.0)
Advantages reported while using technology	
Yes	5 (100.0)
No/not reported	0 (0.0)
Complementary tools or technology	
Laptop	1 (20.0)
Quintic biomechanical analysis software	1 (20.0)
Portable DVD	1 (20.0)
Panasonic AG-7350 recorder, a Sony PVM-1341 monitor and a microcomputer	1 (20.0)
Not reported	1 (20.0)
Used technology reference	
Published	0 (0.0)
Manual	5 (100.0)

We identified 10 studies that updated and/or applied the exact AI-related technology reported in Tables 2 and 3 (Table III, supplemental material). Among those studies, 7/10, (70%) were used for the assessment of motor skills; and 3/10, (30%) were updated and used ( *i.e.*, a new version of the technology).

## Discussion

We performed a scoping review of AI-related technologies developed and used to assess motor skills in children. Engineering work and technological features have been prioritized in these studies, such as the use of advanced motion-capture systems and the training of sophisticated machine-learning algorithms for movement pattern recognition. Validation was predominantly conducted using engineering criteria focused on technical performance, which is appropriate within algorithm-development contexts. However, when these technologies are intended to function as assessment tools for children’s motor skills, additional evidence on measurement properties becomes relevant from a clinical and psychometric perspective. Therefore, our findings suggest an interdisciplinary gap between technical validation and assessment-oriented validation, rather than a deficiency of engineering approaches alone. The use of these AI-related technologies in scientific research is still limited.

Most studies on AI technologies engineering ignored the standard psychometric validation process ( *i.* *e.*, COSMIN standards). Although many of them complied with the good practices in the development of image processing-oriented software, none of them integrated a substantive validation. AI-related technology is good for identifying movement patterns that are rare in children or patterns that children of a certain age should show, and they are not. This capacity has enormous value for clinical and educative purposes. However, for these AI measures to be integrated into a formal clinical evaluation, some technical features must be confirmed. For example, the measurement error estimate is essential for evaluating individuals from the target population, allowing the definition of critical ranges ( *i.e.*, minimum and maximum values) to contrast individual measures and conclude an advantaged, normal or sub-normal motor skill development. Another important psychometric characteristic is responsiveness, which reveals whether any change seen between within-individual AI measurements performed before and after an intervention corresponds to true changes in motor skills (smallest detectable changes), which is linked to investigating when these changes are clinically relevant (minimal important changes).

A previous review of AI technologies for evaluating motor skills in paediatric populations warns that the validation of these tools is limited. ^39^ As we do here, they concluded that this limitation has practical implications in the assessment precision and applicability in clinical contexts. Without a standard psychometric validation process, AI developers do not collect the correct and sufficient evidence to ensure the minimal validity and reliability required for this kind of measurement. For example, one of our reviewed studies reported that the AI algorithm was reliable and valid because it was based on a test previously declared reliable by its original author. ^40^ Differences between the population for which the original test was created and the sample used to develop the AI version can seriously compromise the reliability of the measures and their clinical interpretation criterion due to cultural/ethnic, linguistic, social, economic and age differences. ^10^ In practice, clinical interpretation is an essential component of measurement validity and usually requires evidence beyond the standard qualification norm. For example, the recent study reported a new video-based technology that was based on a classical motor skill test ( *i.e.*, that needs paper, pencil and evaluator’s criteria), showing concurrent validity against another measure of motor skills. ^41^ Contrasting AI measurements against external independent criteria is essential, not only to confirm that the algorithm is measuring what we intend to but also to connect these measurements with other signs and symptoms clinically relevant. In this way, AI measurements become more informative and useful for a full evaluation of a children’s healthy development. ^42^

There are some factors explaining the limited production of AI-related technologies for evaluating motor skills in children. There is a priority for using AI to assess other health problems in this and other populations. During the last two decades, most AI for health has been developed for the diagnosis and follow-up of physical problems such as cancer, cardiovascular diseases, or neurodegenerative disorders in adult subjects. ^18^^,^^43^ High costs slow the production of these AI-related technologies, ^44^^,^^45^ especially in low-and-middle-income countries. Rich countries promote the investment of significant amounts of money for developing new cutting-edge technology, ^46^ although for a wide range of purposes. In low- and middle-income countries, AI development suffers from some extra limitations, such as insufficient economic and human resources, limited data, non-transparent AI algorithm sharing, and scarce collaboration between technological institutions. ^47^

The use of AI-related technologies in scientific research is also limited, and this is linked to other factors. As expected, developers focused on engineering and not research to facilitate the use of their technologies. For example, only one of our reviewed studies performed a usability and feasibility analysis, ^48^ which is important to make the technology friendlier and more accessible to future users. ^40^ This can be explained, in part, because most of them is still developed within the academia, and not yet in the private sector and for commercial purposes. However, considering how they can improve the speed and precision of BMS evaluation of children for doctors and teachers, these AI-related technologies have great commercial potential in the educative and clinical contexts.

### Strengths and Limitations

This is the first scoping review emphasising the substantive validation processes of AI-related technologies produced to assess motor skills in preschool children. The databases consulted during the identification and selection of studies were specialised and extensive; thus, there was a limited loss of relevant information. Also, although this review was based on COSMIN standards to assess the psychometric quality of AI-related technologies, due to the heterogeneity observed in the included studies, no specific adjustments were made to control for possible confounding variables. Therefore, the conclusions need to be interpreted with caution. It is recommended that future research address these factors and use control methods to provide more generalizable conclusions. Furthermore, feasibility and usability were extracted only if the reviewed studies explicitly reported having done so in their analysis of AI-related technologies. Therefore, further studies should evaluate these analyses using a standardized framework. This review did not aim to analyze associations between variables; however, variability in sample sizes, age ranges, and types of AI-based technologies used across studies may affect the comparability and generalizability of the findings. These differences should be considered when interpreting the results and highlight the need for more standardized approaches in future research. The literature search was conducted up to January 30, 2023. Given the rapid evolution of artificial intelligence and computer vision, more recent studies may not have been captured. Therefore, the findings should be interpreted as reflecting the evidence available up to that date rather than the most current state of the field.

### Implications

To facilitate use, developers could conduct studies that evaluate the acceptance, ease of use, cost-effectiveness, and accessibility of these technologies. For example, most technologies rely on sensors and monitors that, while accurate, can be costly, require specialized training, and can be difficult to implement in real-world settings for physicians, teachers, therapists, or practitioners unfamiliar with these tools. In addition, disparities in access to advanced technologies may limit their widespread adoption, particularly in low-resource settings.

Also, these types of technologies may be closer to more universal and cost-effective devices, such as video cameras, smartphones, and tablets, that can assess and report motor skills in real time. However, addressing these challenges requires a collaborative and interdisciplinary approach. At present, there is no universal standardized assessment protocol that adequately bridges clinical psychometric requirements with algorithmic and sensor-based evaluation for AI-related technologies used in BMS assessment. Therefore, future research should move toward an interdisciplinary framework that defines common standards for both measurement properties (e.g., validity, reliability, measurement error, responsiveness) and technical procedures (e.g., data acquisition, calibration, synchronization, and reporting), in order to improve comparability, scalability, and practical implementation across settings. ^50^ Future validation studies should involve experts from multiple fields, including engineers, healthcare professionals, educators and policy makers, to ensure that these technologies are not only accurate, but also practical, scalable and accessible to diverse populations.

New validation studies of these technologies should include validation standards for BMS tests, prioritizing key psychometric properties such as construct validity, criterion validity, reliability, measurement error, among others. To make this possible, engineering teams could incorporate specialists in psychometrics, developmental therapy and medicine to work collaboratively. This multidisciplinary approach will facilitate the integration of medical knowledge and psychometric standards into future software releases, improving both measurement accuracy and practical usability. Finally, developers should consider providing open source code or detailed methodological documentation, which will allow for further refinement, replication, and clinical adaptation of these technologies in future research and real-world applications.

## Conclusions

Engineering work and technological features have been prioritized in the studies about AI-related technologies. The validation of these algorithms was strictly based on engineering criteria; it means, no substantive knowledge of the medical or psychological aspects of motor skills was integrated into the validation process. Technical features typically evaluated in psychometric instruments designed for assessing motor skills in children were also ignored ( *e.g.*, COSMIN criteria). The use of these AI-related technologies in scientific research is still limited.

## Data Availability

Zenodo: Development, validation and use of artificial-intelligence-related technologies to assess basic motor skills in children: a scoping review, https://doi.org/10.5281/zenodo.8056742 ^33^ This project contains the following extended data:
• Appendix 1. Supplementary Tables • Appendix 2. Search formulas Also in Zenodo: Figueroa-Quiñones, Joel. (2023). date extension. Zenodo. https://doi.org/10.5281/zenodo.8190823. ^34^ It’s found:
• Information extraction form Finally, in Zenodo: Joel. (2023). data extension [Data set]. Zenodo. https://doi.org/10.5281/zenodo.8253201. ^31^ This project contains the following extended data:
• 1. COSMIN checklist • 2. Scoping Reviews (PRISMA-ScR) Checklist • 3. Flowchart Data are available under the terms of the Creative Commons Attribution 4.0 International license (CC-BY 4.0).
