# Supplementary material for: Development, validation and use of artificial-intelligence-related technologies to assess basic motor skills in children: a scoping review
Source: F1000Res. 2026 Apr 24;12:1598. Originally published 2023 Dec 18. [Version 3] doi: 10.12688/f1000research.138616.3 (PMC13248934; doi:10.12688/f1000research.138616.3)
Supplement: Peer review history

# Reviewer report: Reviewer response for version 3

**DOI:** 10.5256/f1000research.198263.r488396

**Published:** 2026-06-03

**Authors:** Faisal Dharma Adhinata

**Affiliations:** 1 Telkom University, Purwokerto, Indonesia

This article reviews previous studies related to the use of Artificial Intelligence (AI) to assess basic motor skills in children. The method used for the review process is PRISMA, where the researcher also uses the COSMIN criteria for the instrument diagnosis. The researcher has obtained many articles, but only 12 articles were selected for full text extraction. Then, there is a flow related to the use of AI from input to output as shown in Figure 1. The researcher has not provided examples related to the method according to the stages in Figure 1.

 Here are some suggestions for improving this review article:

 1. Provide examples of the methods used based on the step-by-step division in Figure 1. This example method is based on an article that has been fully reviewed.

 2. The use of AI, such as machine learning and deep learning, has its own strengths and weaknesses. The author has not discussed the strengths and weaknesses of each approach.

 3. Which AI method approach is most suitable for assessing basic motor skills in children? Provide recommendations in the discussion section.

Are the rationale for, and objectives of, the Systematic Review clearly stated?

Yes

Is the statistical analysis and its interpretation appropriate?

I cannot comment. A qualified statistician is required.

If this is a Living Systematic Review, is the ‘living’ method appropriate and is the search schedule clearly defined and justified? (‘Living Systematic Review’ or a variation of this term should be included in the title.)

Yes

Are sufficient details of the methods and analysis provided to allow replication by others?

Partly

Are the conclusions drawn adequately supported by the results presented in the review?

Yes

Reviewer Expertise:

Artificial Intelligence and Computer Vision

I confirm that I have read this submission and believe that I have an appropriate level of expertise to confirm that it is of an acceptable scientific standard, however I have significant reservations, as outlined above.

# Reviewer report: Reviewer response for version 3

**DOI:** 10.5256/f1000research.198263.r478798

**Published:** 2026-04-25

**Authors:** Dhahbi Wissem

**Affiliations:** 1 University of Jendouba, Kef, Tunisia

**General Comments**

 This third version of the scoping review significantly improves upon previous iterations by addressing the interdisciplinary gap between engineering validation and clinical psychometric standards. The authors have successfully refined the manuscript to clarify that the application of COSMIN standards was intended as a measurement-oriented appraisal rather than a critique of algorithmic efficiency. While the temporal limitation of the literature search (cutoff January 30, 2023) remains a constraint, the authors have appropriately framed this as a historical mapping of evidence available at the time of original submission. The manuscript now provides a coherent narrative that bridges the technical capabilities of AI-related technologies with the practical requirements of pediatric motor assessment.

 Specific Comments

**1. Methodological Framing and COSMIN Application**

 The authors have clarified the rationale for using COSMIN guidelines, explicitly stating they were applied to examine evidence relevant to assessment use rather than to evaluate algorithmic performance per se.

 The distinction between engineering validation (focused on accuracy and technical performance) and clinical/psychometric validation (focused on reliability and responsiveness) is now well-defined in the Results and Discussion sections.

 2. Introduction and Contextual Background

 The Introduction now effectively connects early basic motor skill (BMS) competency with long-term biomechanical implications, such as movement efficiency and potentially lower injury risk.

 The revised description of AI pipelines acknowledges modern deep learning-based pipelines and vision transformers, providing a more balanced view of contemporary computational approaches.

 3. Addressing Limitations and Bias

 The authors have explicitly acknowledged the search cutoff date in the Strengths and Limitations section, providing necessary context for the reader to interpret the results within that specific temporal scope.

 Typographical and formatting errors identified in previous versions, such as the "Participant gender" categorization and geographical misspellings, have been corrected.

 4. Implications for Future Research

 The implications section has been strengthened to highlight the urgent need for a universal standardized assessment protocol that bridges the gap between engineering and clinical disciplines.

 The recommendation for interdisciplinary collaboration involving engineers, healthcare professionals, and educators is a critical addition for the practical implementation of these technologies.

Are the rationale for, and objectives of, the Systematic Review clearly stated?

Yes

Is the statistical analysis and its interpretation appropriate?

Not applicable

If this is a Living Systematic Review, is the ‘living’ method appropriate and is the search schedule clearly defined and justified? (‘Living Systematic Review’ or a variation of this term should be included in the title.)

Yes

Are sufficient details of the methods and analysis provided to allow replication by others?

Partly

Are the conclusions drawn adequately supported by the results presented in the review?

Yes

Reviewer Expertise:

AI and Sports Sciences

I confirm that I have read this submission and believe that I have an appropriate level of expertise to confirm that it is of an acceptable scientific standard.

# Reviewer report: Reviewer response for version 2

**DOI:** 10.5256/f1000research.186910.r469885

**Published:** 2026-03-26

**Authors:** Dhahbi Wissem

**Affiliations:** 1 University of Jendouba, Kef, Tunisia

General Comments

 The manuscript presents a scoping review evaluating the development, validation, and use of AI-related technologies for assessing basic motor skills (BMS) in children aged 3 to 6. While the objective of bridging engineering validation with clinical psychometric standards is highly relevant, the paper suffers from critical methodological and temporal flaws.

 Major Weaknesses:

 Outdated Literature Search: The most severe flaw is the search cutoff date of January 30, 2023. For a manuscript undergoing revision in 2025, a >2.5-year gap in a rapidly evolving field like artificial intelligence and computer vision renders the findings almost obsolete. The low prevalence of deep learning applications identified in the results is likely a direct consequence of this outdated search, rather than a true reflection of the current state of the art.

 Misapplication of Evaluation Frameworks: The authors apply the COSMIN checklist—a tool primarily designed for Patient-Reported Outcome Measures (PROMs) and adapted for clinician-reported outcomes—to engineering algorithms. While evaluating the clinical validity of these tools is necessary, computer scientists validate models using benchmark metrics (e.g., Mean Per Joint Position Error, precision, recall, F1-score). The manuscript penalizes engineering papers for not using clinical psychometrics without sufficiently addressing this disciplinary disconnect.

 Limited Sample Size: The scoping review relies on a highly restricted yield of 12 studies (7 for development/validation, 5 for use). Drawing broad conclusions about the entire landscape of AI in pediatric motor assessment from such a limited pool undermines the manuscript's generalizability.

 Minor Weaknesses:

 Typographical and Formatting Errors: Despite being a revised version, several typographical errors persist in the tables and text.

 Over-simplification of AI Pipelines: The description of AI methodologies in the introduction leans heavily on traditional signal processing and classical machine learning, underrepresenting the current dominance of deep learning and vision transformers in pose estimation.

 Specific Comments

 Page 1, Abstract, Methods: The abstract fails to state the cutoff date of the literature search. This is a critical parameter for a scoping review and must be explicitly declared in the abstract.

 Page 4, Introduction, Paragraph 1: The manuscript briefly mentions physical development but fails to connect early motor skill competency with long-term athletic optimization and injury risk mitigation. Expand on the long-term biomechanical implications of early BMS acquisition by referencing recent paradigms: “[Dhahbi W: Advancing biomechanics: enhancing sports performance, mitigating injury risks, and optimizing athlete rehabilitation. In., vol. 7: Frontiers Media SA; 2025: 1556024.]”

 Page 4, Introduction, Paragraph 2: The statistics regarding the prevalence of observer bias (3.2% reporting interobserver reliability; 1.9% meeting rigorous criteria) are supported by citations from 2012 and 2015. Update these figures with contemporary evidence, as methodological reporting standards have evolved over the last decade.

 Page 4, Introduction, Paragraph 3: The explanation of AI pipelines emphasizes "Fast Fourier Transformation," "principal components analysis," and "linear discriminant analysis". These are classical machine learning techniques. While pose estimation (OpenPose) is mentioned, the technical description does not accurately reflect modern deep learning pipelines (e.g., convolutional neural networks or vision transformers) which bypass manual feature extraction entirely.

 Page 6, Search strategy, Paragraph 1: The literature search ended on "January 30, 2023". This is unacceptable for a manuscript published in 2025 focusing on AI. The search must be updated to at least late 2024 to capture the explosion of computer vision models applied to human kinematics.

 Page 7, Data analysis, Paragraph 2: The methodology relies exclusively on COSMIN to evaluate technical quality. The manuscript lacks a robust justification for applying a psychometric tool to algorithmic development papers. Add a methodological disclaimer addressing the fact that engineers evaluate systems using distinct metrics (e.g., accuracy, loss functions) rather than clinical reliability indices.

 Page 8, Table 1: Correct the typographical error "Nort America" to "North America".

 Page 8, Table 1: The category "Participant gender" lists "Both" but includes a sub-category "Just kids". This categorization is illogical and requires reclassification.

 Page 9, Table 2: The reporting of "Motion capture devices" is overly broad and lacks methodological rigor. Detail the calibration and sampling rate issues inherent to combining visual data with biomechanical sensors. To improve the methodological robustness of sensor evaluation, incorporate findings on the methodological constraints of such devices: “[Dhahbi W, Chaouachi A, Cochrane J, Chèze L, Chamari K: Methodological issues associated with the use of force plates when assessing push-ups power. The Journal of Strength & Conditioning Research 2017, 31(7):e74.]”

 Page 9, Table 2: The table reports only 1 study (14.3%) utilizing "Deep learning and neural networks". This finding is highly suspect and strongly indicates that the search strategy or the 2023 cutoff date failed to capture the modern landscape of pose estimation technologies.

 Page 10, Table 3: The application of the COSMIN standards results in almost uniform "Inadequate" or "Doubtful" ratings. Provide clarity in the text on whether these papers failed standard psychometric validation, or if they simply did not attempt it because their primary focus was algorithmic engineering.

 Page 11, Paragraph 1: The manuscript identifies a lack of "responsiveness" and "reliability" reporting (per COSMIN standards) but fails to provide a concrete benchmark of what adequate reporting looks like in physical performance assessment. Clarify this gap by contrasting AI validation with the rigorous intrasession reliability and external responsiveness required in traditional functional motor tests: Refer to reference no. 1

 Page 11, Paragraph 3: The discussion frames the lack of COSMIN adherence as a failure of the developers. Reframe this to acknowledge the interdisciplinary gap: computer science validates via dataset benchmarks, while clinical science requires psychometrics. Both are necessary, but judging one by the standards of the other without context is reductive.

 Page 12, Strengths and Limitations: The authors fail to list the outdated search date (Jan 2023) as a limitation. This must be explicitly stated as a severe threat to the currency and validity of the findings.

 Page 12, Implications: The recommendations for future multidisciplinary approaches are vague. Explicitly address the current absence of, and urgent need for, a universal standardized assessment protocol that bridges clinical psychometrics and algorithmic sensor evaluation. Integrate this call to action by referencing contemporary directives on standardizing AI and sensor technologies in performance assessment: Refer to reference no. 2

Are the rationale for, and objectives of, the Systematic Review clearly stated?

Yes

Is the statistical analysis and its interpretation appropriate?

Not applicable

If this is a Living Systematic Review, is the ‘living’ method appropriate and is the search schedule clearly defined and justified? (‘Living Systematic Review’ or a variation of this term should be included in the title.)

Yes

Are sufficient details of the methods and analysis provided to allow replication by others?

Partly

Are the conclusions drawn adequately supported by the results presented in the review?

Yes

Reviewer Expertise:

AI and Sports Sciences

I confirm that I have read this submission and believe that I have an appropriate level of expertise to confirm that it is of an acceptable scientific standard, however I have significant reservations, as outlined above.

## References

[1] : External responsiveness and intrasession reliability of the rope-climbing test. *The Journal of Strength & Conditioning Research, 30(10), 2952–2958* .2016;10.1519/JSC.000000000000136726849786

[2] : The Algorithmic Athlete: A Call to Standardize Assessment of Sensor Technologies and Artificial Intelligence. *International Journal of Sports Physiology and Performance, 1(aop), 1–2* .2026;10.1123/ijspp.2025-054741493778

# Response

**Published:** 2026-03-30

**Authors:** Joel Figueroa-Quiñones

**Affiliations:** Lambayeque, Universidad Senor de Sipan, Chiclayo, Lambayeque, Peru

Outdated Literature Search: The most severe flaw is the search cutoff date of January 30, 2023. For a manuscript undergoing revision in 2025, a >2.5-year gap in a rapidly evolving field like artificial intelligence and computer vision renders the findings almost obsolete. The low prevalence of deep learning applications identified in the results is likely a direct consequence of this outdated search, rather than a true reflection of the current state of the art.

**We thank the reviewer for this important observation.**

**However, this search date corresponded to the evidence available at the time of the manuscript’s original submission. The extended interval between submission and the present revision was due to the editorial and peer-review process, which was beyond the authors’ control. Therefore, the review should be interpreted as a mapping of the literature available up to January 30, 2023, rather than as a fully updated representation of the field at the time of revision.**

**We have clarified this point in the manuscript and strengthened the limitations section, explicitly acknowledging that more recent studies, including potentially a greater number of deep learning applications, may not have been captured. This issue has been highlighted to ensure an appropriate interpretation of the findings.**

 Misapplication of Evaluation Frameworks: The authors apply the COSMIN checklist—a tool primarily designed for Patient-Reported Outcome Measures (PROMs) and adapted for clinician-reported outcomes—to engineering algorithms. While evaluating the clinical validity of these tools is necessary, computer scientists validate models using benchmark metrics (e.g., Mean Per Joint Position Error, precision, recall, F1-score). The manuscript penalizes engineering papers for not using clinical psychometrics without sufficiently addressing this disciplinary disconnect.

**We respectfully disagree with the view that the COSMIN framework was misapplied in the present review. Our use of COSMIN was not intended to evaluate the engineering performance of artificial intelligence algorithms per se, nor to replace standard computer science benchmarks such as precision, recall, F1-score, or positional error metrics.**

**Rather, COSMIN was used because the focus of this review was on AI-related technologies insofar as they are proposed and used as assessment tools for basic motor skills in children. From this perspective, the key question is not only whether an algorithm performs well computationally, but also whether the resulting tool demonstrates adequate measurement properties for assessment purposes, such as validity, reliability, and interpretability within clinical, educational, or psychomotor contexts.**

**In other words, benchmark metrics may demonstrate technical performance, but they do not by themselves establish that a tool is suitable as a measurement instrument in applied settings. For this reason, we considered COSMIN an appropriate framework to appraise the extent to which the included studies provided evidence relevant to measurement quality.**

**We agree, however, that this involves an interdisciplinary interface between engineering validation and clinical/psychometric evaluation. To avoid misunderstanding, we have clarified in the revised manuscript that COSMIN was applied from a measurement and clinical assessment perspective, not as a substitute for engineering model validation standards. We have also made explicit that the absence of COSMIN-related evidence in some studies should not be interpreted as poor algorithmic quality, but rather as limited reporting of measurement properties relevant to assessment use.**

 Limited Sample Size: The scoping review relies on a highly restricted yield of 12 studies (7 for development/validation, 5 for use). Drawing broad conclusions about the entire landscape of AI in pediatric motor assessment from such a limited pool undermines the manuscript's generalizability.

**We respectfully disagree that the inclusion of 12 studies inherently undermines the contribution of the review. The purpose of a scoping review is not to generate statistically generalizable conclusions about an entire field, but rather to map, characterize, and synthesize the nature and extent of the available evidence on a given topic.**

**In this sense, the limited number of eligible studies should not be interpreted solely as a weakness of the review, but also as a reflection of the current state of the literature at the intersection of artificial intelligence and pediatric basic motor skill assessment. The restricted yield suggests that this remains an emerging and still relatively fragmented area of research, particularly when applying predefined eligibility criteria focused on development, validation, and practical use in children.**

**Importantly, our manuscript does not intend to overgeneralize the evidence base. Instead, the conclusions are framed to reflect the scope and volume of the studies identified, highlighting trends, methodological characteristics, and existing gaps in the literature. Therefore, the small number of included studies is itself a relevant finding, as it underscores the need for further research in this field.**

 Minor Weaknesses:

 Typographical and Formatting Errors: Despite being a revised version, several typographical errors persist in the tables and text.

**We acknowledge that some typographical and formatting inconsistencies remained in the revised version. In response, we carefully reviewed the entire manuscript and corrected the identified errors in the text and tables to improve clarity, consistency, and overall presentation.**

 Over-simplification of AI Pipelines: The description of AI methodologies in the introduction leans heavily on traditional signal processing and classical machine learning, underrepresenting the current dominance of deep learning and vision transformers in pose estimation.

**We agree that recent advances in artificial intelligence for pose estimation have increasingly been driven by deep learning approaches, including convolutional neural networks and, more recently, transformer-based architectures.**

**However, the purpose of the Introduction was not to provide an exhaustive technical review of the full evolution of AI pipelines, but rather to offer a general conceptual background for readers from clinical, developmental, and assessment-oriented fields. In this context, we described traditional signal processing and classical machine learning approaches because they remain part of the methodological foundation of the field and are also represented in the body of studies included in our review.**

**Moreover, the emphasis in the manuscript is on the application of AI-related technologies for the assessment of basic motor skills in children, rather than on benchmarking the latest computer vision architectures. Therefore, the introductory section was intentionally oriented toward framing the field in accessible and applied terms.**

 Specific Comments

 Page 1, Abstract, Methods: The abstract fails to state the cutoff date of the literature search. This is a critical parameter for a scoping review and must be explicitly declared in the abstract.

**We agree that the literature search cutoff date is a critical parameter in a scoping review and should be explicitly stated in the abstract. In response, we revised the Methods section of the abstract to indicate that the search included studies published up to January 30, 2023.**

 Page 4, Introduction, Paragraph 1: The manuscript briefly mentions physical development but fails to connect early motor skill competency with long-term athletic optimization and injury risk mitigation. Expand on the long-term biomechanical implications of early BMS acquisition by referencing recent paradigms: “[Dhahbi W: Advancing biomechanics: enhancing sports performance, mitigating injury risks, and optimizing athlete rehabilitation. In., vol. 7: Frontiers Media SA; 2025: 1556024.]”

**We agree that the long-term biomechanical relevance of early basic motor skill (BMS) development could be stated more explicitly in the Introduction. Although the present review focuses on the assessment of BMS in preschool children rather than on athletic performance per se, early motor competence may have broader biomechanical implications across the life course, including movement efficiency, later participation in physical and sport activities, and potentially injury-risk mitigation through the development of more coordinated and stable movement patterns.**

**In response, we revised the first paragraph of the Introduction to better connect early BMS acquisition with its longer-term biomechanical relevance and to acknowledge recent perspectives highlighting the role of biomechanics in performance optimization, injury-risk reduction, and rehabilitation.**

 Page 4, Introduction, Paragraph 2: The statistics regarding the prevalence of observer bias (3.2% reporting interobserver reliability; 1.9% meeting rigorous criteria) are supported by citations from 2012 and 2015. Update these figures with contemporary evidence, as methodological reporting standards have evolved over the last decade.

**We thank the reviewer for this observation. We acknowledge that the cited prevalence figures were derived from earlier reviews and may not reflect current reporting practices. However, these references were retained because they provide direct quantitative evidence of longstanding concerns regarding interobserver reliability and observer-related bias in behavioral research. To avoid implying that these figures represent the current prevalence, we revised the text to frame them as historical evidence of a methodological problem rather than as a contemporary estimate.**

 Page 4, Introduction, Paragraph 3: The explanation of AI pipelines emphasizes "Fast Fourier Transformation," "principal components analysis," and "linear discriminant analysis". These are classical machine learning techniques. While pose estimation (OpenPose) is mentioned, the technical description does not accurately reflect modern deep learning pipelines (e.g., convolutional neural networks or vision transformers) which bypass manual feature extraction entirely.

**We agree that the original wording of this paragraph placed greater emphasis on classical machine-learning pipelines, which may not fully reflect the diversity of contemporary AI approaches. However, our intention was not to suggest that all current motion-analysis systems rely on manual feature extraction, but rather to provide a broad and accessible description of computational steps that may be involved in some AI-related technologies for human movement assessment.**

**To address this concern, we revised the paragraph to clarify that, although some approaches still involve preprocessing, feature extraction, and conventional classification procedures, many contemporary pipelines rely on end-to-end deep learning models, such as convolutional neural networks and vision transformers, which can learn relevant representations directly from images, videos, or pose sequences with limited or no manual feature extraction. This clarification has been incorporated to better reflect the current methodological landscape.**

 Page 6, Search strategy, Paragraph 1: The literature search ended on "January 30, 2023". This is unacceptable for a manuscript published in 2025 focusing on AI. The search must be updated to at least late 2024 to capture the explosion of computer vision models applied to human kinematics.

**We appreciate the reviewer’s concern and agree that the pace of development in AI-related research is particularly rapid. The literature search in this review ended on January 30, 2023 because this date corresponded to the original submission stage of the manuscript. The prolonged interval before the current revision was largely attributable to the editorial and peer-review timeline, which was outside the authors’ control.**

**Accordingly, the review is intended to represent the evidence available up to that date, rather than the current state of the art at the time of revision. To ensure appropriate interpretation, we have clarified this point in the manuscript and strengthened the limitations section, explicitly acknowledging that newer studies on computer vision models applied to human movement assessment may not have been captured.**

 Page 7, Data analysis, Paragraph 2: The methodology relies exclusively on COSMIN to evaluate technical quality. The manuscript lacks a robust justification for applying a psychometric tool to algorithmic development papers. Add a methodological disclaimer addressing the fact that engineers evaluate systems using distinct metrics (e.g., accuracy, loss functions) rather than clinical reliability indices.

**We agree that engineering studies commonly evaluate systems using discipline-specific performance metrics rather than clinical reliability indices. To clarify our methodological approach, we added a brief statement in the Data analysis section indicating that COSMIN was applied from a measurement perspective to examine evidence relevant to assessment use, rather than to evaluate algorithmic performance per se.**

 Page 8, Table 1: Correct the typographical error "Nort America" to "North America".

**The word was updated**

 Page 8, Table 1: The category "Participant gender" lists "Both" but includes a sub-category "Just kids". This categorization is illogical and requires reclassification.

**We agree that the category “Just kids” was unclear and inappropriate under the variable “Participant gender.” This was a wording error. We revised Table 1 to use clearer and logically consistent categories: “Boys only,” “Girls only,” “Both boys and girls,” and “Not reported.”**

 Page 9, Table 2: The reporting of "Motion capture devices" is overly broad and lacks methodological rigor. Detail the calibration and sampling rate issues inherent to combining visual data with biomechanical sensors. To improve the methodological robustness of sensor evaluation, incorporate findings on the methodological constraints of such devices: “[Dhahbi W, Chaouachi A, Cochrane J, Chèze L, Chamari K: Methodological issues associated with the use of force plates when assessing push-ups power. The Journal of Strength & Conditioning Research 2017, 31(7):e74.]”

**We agree that device-related factors such as calibration procedures, sampling rate, and synchronization may influence motion analysis results. However, the purpose of Table 2 was to provide a descriptive summary of the motion-capture devices and AI-related tools reported in the included studies, rather than to conduct a device-specific technical appraisal of biomechanical sensor performance.**

**We respectfully note that the reference suggested by the reviewer addresses methodological issues related to force plates in the assessment of push-up power, which corresponds to a different biomechanical and performance-testing context from the present review, focused on AI-related technologies for assessing basic motor skills in children. For this reason, we did not incorporate that reference directly into Table 2.**

 Page 9, Table 2: The table reports only 1 study (14.3%) utilizing "Deep learning and neural networks". This finding is highly suspect and strongly indicates that the search strategy or the 2023 cutoff date failed to capture the modern landscape of pose estimation technologies.

**We agree that deep learning approaches have become increasingly prominent in the broader pose estimation literature. However, we respectfully note that the proportion reported in Table 2 reflects only the characteristics of the studies included in this review, based on the predefined eligibility criteria and review period, rather than the overall prevalence of deep learning approaches across the wider field. Therefore, the finding should be interpreted within the specific scope of this scoping review and not as a comprehensive representation of the current pose estimation landscape.**

 Page 10, Table 3: The application of the COSMIN standards results in almost uniform "Inadequate" or "Doubtful" ratings. Provide clarity in the text on whether these papers failed standard psychometric validation, or if they simply did not attempt it because their primary focus was algorithmic engineering.

**We agree that the predominance of “Inadequate” and “Doubtful” ratings in Table 3 requires careful interpretation. These ratings were intended to reflect the extent to which the included studies reported psychometric or measurement-related evidence according to COSMIN standards, rather than their algorithmic or engineering quality per se. In many cases, the included papers primarily focused on technical or algorithmic development, which likely explains the predominance of lower COSMIN ratings. We clarified this interpretive point in the text accompanying Table 3.**

 Page 11, Paragraph 1: The manuscript identifies a lack of "responsiveness" and "reliability" reporting (per COSMIN standards) but fails to provide a concrete benchmark of what adequate reporting looks like in physical performance assessment. Clarify this gap by contrasting AI validation with the rigorous intrasession reliability and external responsiveness required in traditional functional motor tests: Refer to reference no. 1

**We agree that the original text did not sufficiently clarify what adequate evidence of reliability and responsiveness would look like when these AI-related technologies are intended to function as assessment tools. In traditional physical performance and functional motor assessment, reliability is typically supported by evidence such as test–retest or intrasession stability, together with indicators of measurement consistency and error. Likewise, responsiveness requires evidence that an instrument can detect meaningful changes in performance over time or after intervention. In contrast, many of the studies included in our review primarily emphasized algorithmic or technical performance and did not report this type of measurement-oriented evidence. We have clarified this point in the manuscript to better explain that the COSMIN-related gap does not simply refer to missing psychometric terminology, but to the limited demonstration of whether these AI-related tools can provide stable and change-sensitive measurements comparable to those expected from established motor assessment instruments.**

 Page 11, Paragraph 3: The discussion frames the lack of COSMIN adherence as a failure of the developers. Reframe this to acknowledge the interdisciplinary gap: computer science validates via dataset benchmarks, while clinical science requires psychometrics. Both are necessary, but judging one by the standards of the other without context is reductive.

**We agree that the original wording of this paragraph may have overstated the interpretation of the COSMIN findings. Our intention was not to suggest that the included studies failed simply because they did not follow psychometric validation standards, but rather to highlight an interdisciplinary gap between engineering-oriented validation and clinical or assessment-oriented validation. In many of the included studies, validation was primarily conducted using engineering criteria focused on technical performance. This is appropriate within computer science and algorithm-development contexts. However, when these technologies are proposed as tools for assessing motor skills in children, additional evidence on measurement properties becomes relevant from a clinical and psychometric perspective. We have revised the paragraph to better acknowledge that both forms of validation are important and complementary, and that the absence of psychometric evidence should not be interpreted as poor engineering quality per se.**

 Page 12, Strengths and Limitations: The authors fail to list the outdated search date (Jan 2023) as a limitation. This must be explicitly stated as a severe threat to the currency and validity of the findings.

**We agree that the search cutoff date represents an important limitation in a rapidly evolving field such as artificial intelligence and computer vision. In response, we explicitly added this issue to the Strengths and Limitations section, stating that the literature search was conducted up to January 30, 2023 and that, consequently, more recent studies may not have been captured. We also clarified that the findings should be interpreted as reflecting the evidence available up to that date rather than the most current state of the field.**

 Page 12, Implications: The recommendations for future multidisciplinary approaches are vague. Explicitly address the current absence of, and urgent need for, a universal standardized assessment protocol that bridges clinical psychometrics and algorithmic sensor evaluation. Integrate this call to action by referencing contemporary directives on standardizing AI and sensor technologies in performance assessment: Refer to reference no. 2

**We agree that the implications section should more explicitly state the current absence of a universal standardized protocol integrating clinical psychometrics with algorithmic and sensor-based evaluation.**

**In response, we revised the implications section to emphasize that future research should move toward a standardized interdisciplinary framework specifying not only psychometric requirements (e.g., validity, reliability, measurement error, responsiveness) but also minimum technical requirements for sensor- and algorithm-based systems, such as data acquisition procedures, calibration, synchronization, and reporting standards.**

**We also strengthened the call for collaboration across engineering, clinical, educational, and psychometric disciplines so that AI-related technologies for BMS assessment can be developed and validated under a more unified and transferable framework.**

# Reviewer report: Reviewer response for version 1

**DOI:** 10.5256/f1000research.151828.r349533

**Published:** 2025-01-30

**Authors:** Abraham M. Joshua, Ashish John Prabhakar

**Affiliations:** 1 Department of Physiotherapy, Kasturba Medical College, Mangalore, Manipal Academy of Higher Education, Manipal, Karnataka, India

**ABSTRACT**

**Background**    -   Redundant phrasing: "two observersers" is misspelled and repetitive.

**Results**             -   Tracking of "10 other  publications" is unclear and vague.

                           -   Overuse of technical language without explanation for general readers (e.g., "engineering criteria").

**Keywords**       -   Missing potential keywords like "assessment tools," “functional abilities,”   

 and ****"gross motor” based on the ‘title and the contents of the study

**Introduction: suggestions**

**1. Overly Technical Language:**

- While the introduction is generally informative, it becomes very technical in certain sections, especially when describing the AI process (e.g., "Fast Fourier Transformation," "principal components analysis," "linear discriminant analysis"). This might be difficult for readers unfamiliar with these terms or the AI field.
- **Suggestion**: Simplify the explanation or provide brief definitions or context for these terms.

**2. Lack of Clear Connection Between BMS and AI:**

- While the introduction discusses BMS, AI technologies, and their potential to address observer bias, the connection between these topics could be more explicit. The introduction seems to jump between BMS, traditional measurement tools, observer bias, and AI without a smooth flow that ties everything together.
- **Suggestion**: Strengthen the connection between the problems with current BMS assessment and how AI could specifically address them. A clearer explanation of how AI can solve observer bias and improve accuracy in BMS assessment would help make the argument more compelling.

**3. Limited Emphasis on the Scope of the Problem:**

- The issue of observer bias and its impact on BMS assessment is raised, but it’s not explored in-depth. The extent of the problem (how often it happens, how significant the impact is) isn’t fully explained.
- **Suggestion**: Provide more concrete examples or data to highlight the real-world implications of observer bias in BMS assessments. This would help emphasize the need for better solutions like AI.

**4. Vague Description of "AI-Related Technologies":**

- The term “AI-related technologies” is introduced, but it remains somewhat vague. While the steps of motion recognition are detailed, it’s not entirely clear what specific AI tools or algorithms are most effective in this context or how these methods directly translate to motor skill assessment in children.
- **Suggestion**: Clarify the specific AI tools used or might be used in BMS assessment. This could make the introduction feel more grounded in practical applications.

**5. Inconsistent Flow and Structure:**

- The flow between sections could be smoother. For example, the transition from talking about BMS instruments (TGMD-2, BOT-2) to observer bias is a bit abrupt. Similarly, after discussing the issue of observer bias, the leap to the technicalities of AI feels disconnected.
- **Suggestion**: Reorganize the content so that each section builds more naturally on the previous one. Consider adding a paragraph that ties observer bias to the introduction of AI as a solution before jumping into the technical aspects.

**6. Missing Emphasis on the Target Age Group (3-6 Years):**

- The introduction discusses BMS in general terms but doesn’t emphasize the importance of assessing motor skills in children aged 3 to 6 years. Since this is a specific target group, more focus on why this age range is critical for BMS development would strengthen the relevance of the study.
- **Suggestion**: Provide a brief explanation of why this age group is the focus and what makes BMS development particularly crucial at this stage.

**7. Lack of Citations for Claims About AI in Other Fields:**

- There are a few claims about AI applications in other fields (e.g., assessing physical activity in adults, recognizing walking alterations), but not all of these are fully supported by citations. The mention of “a recent review” and other general statements could be more precise.
- **Suggestion**: Provide specific references or clarify that the claims about AI in other fields are based on the literature review cited earlier.

**8. No Clear Statement of the Research Gap:**

- While the introduction implies a gap in the literature (i.e., a lack of formal review of AI in BMS assessment for children), this could be more explicitly stated and emphasized. It could be clearer why this review is necessary and what new insights it might offer.
- **Suggestion**: Make the research gap more prominent by rephrasing the last couple of sentences to more forcefully explain why this scoping review is essential.

**9. Repetitive Mention of AI:**

- The introduction repeatedly mentions “AI-related technologies,” which could be streamlined. The term is used several times in close proximity without adding new information each time, which can feel redundant.
- **Suggestion**: Try to vary the phrasing (e.g., "machine learning tools," "AI classifiers") to keep the introduction engaging and avoid repetition.

**10. Unfinished Sentence in Objective Statement:**

- The final sentence of the introduction (before the objectives) seems cut off. This makes the last thought feel incomplete and leaves the reader hanging.
- **Suggestion**: Complete the sentence and ensure that all points are concluded before introducing the research objectives.

**Methods and discussion:**

**1. Lack of Detail in Study Selection Criteria**

- While the article clearly defines the types of studies it targeted (engineering, substantive validation, and use of AI-related technologies), it doesn’t provide much detail on what specific inclusion and exclusion criteria were applied beyond these categories. For example, were studies excluded for reasons like sample size, study design, or methodological rigor?
- **Suggestion:** Provide more specific inclusion and exclusion criteria. Were only randomized controlled trials considered? Were there any restrictions based on the publication type (e.g., peer-reviewed articles only)?

**2. Vague Explanation of Search Strategy**

- The search strategy mentions specific databases but does not explain the rationale for selecting these particular sources over others. Are there other databases relevant to the field that might have been excluded? How were the keywords selected, and were any synonyms or related terms considered?
- **Suggestion:** Provide a clearer justification for the selection of these particular databases and search terms. Did you consider any grey literature (e.g., theses, dissertations, reports from non-peer-reviewed sources) to broaden the search?

**3. Details on the Rayyan Platform**

- The mention of the Rayyan platform for managing studies is helpful, but the text doesn't clarify whether Rayyan was used for the full review process or just the initial screening. The review process appears to be manual in nature, but it could benefit from some details on how disagreements were handled between reviewers (e.g., was there a consensus meeting or did one reviewer have final say on the study’s inclusion?).
- **Suggestion:** Provide more detail on how Rayyan was used throughout the review process. How were disagreements between the two independent groups resolved, and what role did the principal investigator play in the final decisions?

**4. Potential Bias in Study Selection**

- The method states that the initial review of titles and abstracts was done by two independent groups, but there is no mention of any specific strategies to minimize bias during this phase. Given that the authors are likely familiar with the topic, it could be helpful to acknowledge how any biases in study selection (e.g., confirmation bias or publication bias) were minimized.
- **Suggestion: **Mention strategies to reduce bias in study selection. For example, were studies randomly assigned to reviewers, and was there a protocol to ensure that preconceived notions didn’t influence the selection process?

**5. Limited Details on Data Extraction Process**

- While the data extraction form is outlined with categories such as general information, engineering, substantive validation, and use, there is little detail on how the data were extracted from the studies. For example, was any kind of reliability check conducted between reviewers, or were discrepancies resolved through discussion?
- **Suggestion:** Provide more details on the data extraction process, such as whether two independent reviewers performed the extraction and how discrepancies were resolved. Additionally, were any statistical methods used to assess inter-rater reliability in the extraction process?

**6. Lack of Clarity on Data Analysis**

- The description of data analysis mentions the use of descriptive statistics (frequencies and percentages) and the COSMIN standards for evaluating psychometric properties. However, there is no clear explanation of how the data were analyzed or how the results were synthesized. Were any meta-analysis or qualitative synthesis methods considered? How were the studies compared and summarized?
- **Suggestion: **Provide more clarity on how the data were synthesized. Was any quantitative or qualitative analysis beyond simple descriptive statistics performed, such as meta-analysis or thematic analysis? Did you perform a subgroup analysis for specific types of AI technologies or specific study characteristics?

**7. Lack of Discussion on Potential Confounding Variables**

- There is a brief mention of the use of psychometric standards (COSMIN) to evaluate AI technologies, but the article doesn't discuss how confounding factors (e.g., differences in sample size, age groups, or types of technology used) might impact the validity of the conclusions. Were these factors taken into consideration in the evaluation process?
- **Suggestion:** Acknowledge potential confounding variables and how they might affect the validity of the studies reviewed. How did the authors control for these variables, if at all, in the synthesis process?

**8. Unclear Rationale for Data Collection Methods**

- In the section about data extraction, the mention of "feasibility and usability" as part of the data form is important but lacks further context. Was there a specific framework used to evaluate these factors? Were the usability and feasibility criteria standardized across the included studies?
- **Suggestion:** Provide more information on the frameworks or criteria used to assess usability and feasibility. Were these evaluations subjective or based on standardized measures? Clarifying this would help readers understand how the data extraction process assessed these aspects.
- **Discussion**   -    Discuss usability, cost-effectiveness, and accessibility challenges. -Emphasize the need for interdisciplinary collaboration in future validation  studies. -Propose specific psychometric properties (e.g., construct validity) to prioritize  future AI validation research. - Add practical recommendations for improving AI tools (e.g., integrating psychometric standards, improving usability for non-specialists).

Are the rationale for, and objectives of, the Systematic Review clearly stated?

Yes

Is the statistical analysis and its interpretation appropriate?

Partly

If this is a Living Systematic Review, is the ‘living’ method appropriate and is the search schedule clearly defined and justified? (‘Living Systematic Review’ or a variation of this term should be included in the title.)

Yes

Are sufficient details of the methods and analysis provided to allow replication by others?

Partly

Are the conclusions drawn adequately supported by the results presented in the review?

Yes

Reviewer Expertise:

Neurorehabilitation, Upper limb functional evaluation,  AI for rehabilitation, Exercise therapy, Balance training

We confirm that we have read this submission and believe that we have an appropriate level of expertise to confirm that it is of an acceptable scientific standard, however we have significant reservations, as outlined above.

# Response

**Published:** 2025-08-19

**Authors:** Joel Figueroa-Quiñones

**Affiliations:** Lambayeque, Universidad Senor de Sipan, Chiclayo, Lambayeque, Peru

Reviewer # 1:

**Abstract**

 Background    -   Redundant phrasing: "two observersers" is misspelled and repetitive.

**We corrected the typographical error and improved the phrasing for clarity in the Background section. The revised sentence now reads:**

***“In basic motor skills evaluation, two observers may rate the same child’s performance differently, introducing variability in the assessment”***

 Results             -   Tracking of "10 other publications" is unclear and vague.

**We revised the sentence in the Results section to provide a more precise description of the process. The updated text now reads:**

***“From the 7 technology development studies, we examined their citation networks using Google Scholar and identified 10 subsequent peer-reviewed publications that either enhanced the original technologies or applied them in new research contexts.*** *”*

                           -   Overuse of technical language without explanation for general readers (e.g., "engineering criteria").

**We revised the sentence to reduce technical jargon and make it more accessible to general readers. In the corresponding section, the updated sentence now reads:**

***“The validation of these algorithms was based on engineering standards, focusing on their accuracy and technical performance, but without integrating medical and psychological knowledge about children's motor development.”***

 Keywords       -   Missing potential keywords like "assessment tools," “functional abilities,” and ****"gross motor” based on the ‘title and the contents of the study

**We have updated the list of keywords to better reflect the title and content of the study. The following terms have been added:**

***“assessment tools,” “functional abilities,” and “gross motor.”***

 Introduction: suggestions

  

1. Overly Technical Language:

While the introduction is generally informative, it becomes very technical in certain sections, especially when describing the AI process (e.g., "Fast Fourier Transformation," "principal components analysis," "linear discriminant analysis"). This might be difficult for readers unfamiliar with these terms or the AI field.

 Suggestion: Simplify the explanation or provide brief definitions or context for these terms.

**We have revised the third paragraph of the Introduction to provide brief definitions and clearer context for the mentioned techniques. Specifically, we now explain the role of Fast Fourier Transformation, principal components analysis, and linear discriminant analysis in simpler terms to aid reader understanding. The revised sentence reads as follows:**

***“Then, these data undergo pre-processing to reduce noise and enhance relevant features. This step often involves filtering techniques, such as Fast Fourier Transformation (which helps separate important movement signals from background noise) or wavelet transforms. Additionally, to simplify complex data and highlight key movement patterns, methods like principal components analysis (which reduces data dimensions while preserving essential information) or linear discriminant analysis (which enhances the distinction between movement categories) are applied (17).”***

 2. Lack of Clear Connection Between BMS and AI:

 While the introduction discusses BMS, AI technologies, and their potential to address observer bias, the connection between these topics could be more explicit. The introduction seems to jump between BMS, traditional measurement tools, observer bias, and AI without a smooth flow that ties everything together.

 Suggestion: Strengthen the connection between the problems with current BMS assessment and how AI could specifically address them. A clearer explanation of how AI can solve observer bias and improve accuracy in BMS assessment would help make the argument more compelling.

**We agree that the connection between BMS, traditional measurement tools, observer bias, and artificial intelligence (AI)-related technology could be more explicit. In response to your suggestion, we have made adjustments to the third paragraph of the Introduction to improve the flow and strengthen the connection between these topics.**

**Specifically, we added a clearer explanation of how AI can directly address observer bias and improve accuracy in BMS assessment. We now describe how AI, by automating the motion classification process, reduces human subjectivity and enables more objective and accurate evaluations.**

**In this way, we aim to reinforce the argument that AI-related technology not only offers an alternative to traditional methods but effectively addresses the limitations of current BMS assessment approaches.**

 3. Limited Emphasis on the Scope of the Problem:

 The issue of observer bias and its impact on BMS assessment is raised, but it’s not explored in-depth. The extent of the problem (how often it happens, how significant the impact is) isn’t fully explained.

 Suggestion: Provide more concrete examples or data to highlight the real-world implications of observer bias in BMS assessments. This would help emphasize the need for better solutions like AI.

**We have strengthened the second paragraph of the Introduction by incorporating concrete data that illustrate the prevalence and real-world consequences of this issue. Specifically, we now include evidence from two reviews: one indicating that, among 960 behavioral studies, only 3.2% reported interobserver reliability measures and only 1.9% met rigorous criteria for minimizing bias (12); and another highlighting that in child development research, poor reporting and variability in assessor performance may obscure children’s true developmental status, potentially compromising clinical decisions (13).**

**These additions aim to clarify the extent and significance of observer bias, reinforcing the necessity of adopting more objective tools—such as AI-based technologies—for accurate and reliable BMS assessment.**

 4. Vague Description of "AI-Related Technologies":

 The term “AI-related technologies” is introduced, but it remains somewhat vague. While the steps of motion recognition are detailed, it’s not entirely clear what specific AI tools or algorithms are most effective in this context or how these methods directly translate to motor skill assessment in children.

 Suggestion: Clarify the specific AI tools used or might be used in BMS assessment. This could make the introduction feel more grounded in practical applications.

**We have revised the beginning of the third paragraph of the Introduction to explicitly define “AI-related technologies” and specify the tools most relevant for BMS assessment. The revised text reads as follows:**

***“AI-related technologies (i.e., computational systems that use artificial intelligence to analyze, learn from, and interpret data) offer a promising alternative to minimize observer bias in BMS assessment (14). For example, for motion capture and analysis, computer vision tools such as OpenPose, MediaPipe and DeepLabCut enable pose estimation and tracking of key points of the human body with high accuracy (15). In addition, deep learning techniques, such as Convolutional Neural Networks (CNN) and vision-specialized Transformer Models (ViT), have proven to be effective in classifying motion sequences in videos (16). In that sense, these AI-related technologies for recognizing and classifying human motion patterns consist of several steps (Figure 1) (17).”***

 5. Inconsistent Flow and Structure:

 The flow between sections could be smoother. For example, the transition from talking about BMS instruments (TGMD-2, BOT-2) to observer bias is a bit abrupt. Similarly, after discussing the issue of observer bias, the leap to the technicalities of AI feels disconnected.

 Suggestion: Reorganize the content so that each section builds more naturally on the previous one. Consider adding a paragraph that ties observer bias to the introduction of AI as a solution before jumping into the technical aspects.

**We appreciate your feedback on the flow and structure of the manuscript. Our team has improved the text to facilitate smooth transitions between sections.**

**Specifically, the first part of the paragraph now reads: " *Typically, BMS assessment relies on trained professionals who observe, record, and score children's performance on specific motor tasks (8,9). However, a major challenge in this approach is observer bias. Even when raters receive standardized training, small differences in scoring can introduce variability in BMS measurements. This variability reduces the accuracy of the assessment and can lead to misinterpretations. For example, two children with similar motor skills may receive different scores depending on the assessor, resulting in inconsistent results. When these inconsistencies follow a systematic pattern, they contribute to observer bias, a well-documented source of measurement error (10,11)*.".**

**We have also added a transitional paragraph at the end of the second paragraph, linking the problem of observer bias to the introduction of AI technologies as a solution. This paragraph highlights how observer bias can affect BMS assessment results, and the following paragraph addresses how AI-related technologies can mitigate this problem, offering a more objective and accurate alternative. The text reads: *"In fact, one review reported that of 960 behavioral studies, only 3.2% reported measures of interobserver reliability, and only 1.9% met rigorous criteria for minimizing bias (12). Similarly, another review on child development found that the quality of reporting on the use of assessors in these studies was poor and that variability in assessor performance may obscure the true developmental status of children, compromising complex and costly clinical decisions (13)”.***

 6. Missing Emphasis on the Target Age Group (3-6 Years):

 The introduction discusses BMS in general terms but doesn’t emphasize the importance of assessing motor skills in children aged 3 to 6 years. Since this is a specific target group, more focus on why this age range is critical for BMS development would strengthen the relevance of the study.

 Suggestion: Provide a brief explanation of why this age group is the focus and what makes BMS development particularly crucial at this stage.

****

**We have added a brief explanation in the first paragraph of the introduction that highlights why this age group is crucial for BMS development. Specifically, we have pointed out that during this period of rapid physical and motor development, children refine fundamental skills that are essential for later complex activities, which directly impacts their cognitive, emotional, and social development. In addition, we underscored the relevance of early assessment to identify potential delays and facilitate timely interventions.**

**Now the text says the following: *"The development of basic motor skills (BMS) in children aged 3 to 6 years is critical, as this is a period of rapid motor growth, where children acquire physical skills that allow them to participate in a variety of activities (1,2). At this age, children experience significant improvements in gross motor control, allowing them to perform movements such as running, jumping, and manipulating objects with greater precision (3,4). The acquisition of these motor skills is essential for physical, cognitive and emotional development, as BMS are strongly linked to general well-being, self-esteem and social integration (5)."***

 7. Lack of Citations for Claims About AI in Other Fields:

 There are a few claims about AI applications in other fields (e.g., assessing physical activity in adults, recognizing walking alterations), but not all of these are fully supported by citations. The mention of “a recent review” and other general statements could be more precise.

 Suggestion: Provide specific references or clarify that the claims about AI in other fields are based on the literature review cited earlier.

**We have carefully reviewed the claims about AI applications and ensured that each is supported by an appropriate reference. In addition, we have revised the general claims to improve their accuracy with the cited literature.**

**Specifically, we have updated citations 4, 5, 6, and 7 with their respective references:**

**4. Jiang G-P, Jiao X-B, Wu S-K, Ji Z-Q, Liu W-T, Chen X, et al. Balance, proprioception, and gross motor development of Chinese children aged 3 to 6 years. J Mot Behav. 2018;50(3):343–52. Available at: http://dx.doi.org/10.1080/00222895.2017.1363694**

**5. Gandotra A, Kotyuk E, Bizonics R, Khan I, Petánszki M, Kiss L, et al. An exploratory study of the relationship between motor skills and indicators of cognitive and socio-emotional development in preschoolers. Eur J Dev Psychol. 2023;20(1):50–65. Available at: http://dx.doi.org/10.1080/17405629.2022.2028617**

**6. Bremer E, Cairney J. Fundamental Movement Skills and Health-Related Outcomes: A Narrative Review of Longitudinal and Intervention Studies Targeting Typically Developing Children. Am J Lifestyle Med. 2018;12(2):148-59. https://doi.org/10.1177/1559827616640196**

**7. Eddy LH, Wood ML, Shire KA, Bingham DD, Bonnick E, Creaser A, et al. A systematic review of randomized and case-controlled trials investigating the effectiveness of school-based motor skill interventions in 3- to 12-year-old children. Child Care Health Dev. 2019;45(6):773-90. https://doi.org/10.1111/cch.12712**

 8. No Clear Statement of the Research Gap:

 While the introduction implies a gap in the literature (i.e., a lack of formal review of AI in BMS assessment for children), this could be more explicitly stated and emphasized. It could be clearer why this review is necessary and what new insights it might offer.

 Suggestion: Make the research gap more prominent by rephrasing the last couple of sentences to more forcefully explain why this scoping review is essential.

**We have revised the last two final sentences of the introduction to highlight the research gap in the use of AI to accurately assess BMS in children and the need for this scoping review. Specifically, we now emphasize the lack of a comprehensive review of AI applications for assessing basic motor skills (BMS) in preschool children, as well as the need to systematize the scope, limitations, and validity of these technologies.**

**Specifically, we have added the following: *"However, despite the increasing use of AI in motor performance assessment, there is no comprehensive review examining its specific application in the assessment of BMS in preschool children, being a crucial stage for early detection and intervention. Furthermore, the scope, limitations and validity of AI-based technologies in this context are not yet clearly systematized. Therefore, it is required to synthesize existing knowledge and guide the development of more accurate and accessible assessment tools"***

 9. Repetitive Mention of AI:

 The introduction repeatedly mentions “AI-related technologies,” which could be streamlined. The term is used several times in close proximity without adding new information each time, which can feel redundant.

 Suggestion: Try to vary the phrasing (e.g., "machine learning tools," "AI classifiers") to keep the introduction engaging and avoid repetition.

**Our team has made sure to review and update the text to avoid redundancy of the term “AI-related technologies”. We have also decided to keep the term “AI-related technologies” consistently throughout the introduction to ensure clarity and consistency throughout the manuscript. In fact, in the third paragraph, we have included a clarification referring to the term “AI-related technologies”.**

**Specifically, in the first three lines of the third paragraph in the introduction, we have added: *"AI-related technologies (i.e., computational systems that use artificial intelligence to analyze, learn from, and interpret data) offer a promising alternative to minimize observer bias in BMS assessment (14)"***

 10. Unfinished Sentence in Objective Statement:

 The final sentence of the introduction (before the objectives) seems cut off. This makes the last thought feel incomplete and leaves the reader hanging.

 Suggestion: Complete the sentence and ensure that all points are concluded before introducing the research objectives.

**We have reviewed and revised this section to ensure the idea is fully expressed and concluded before presenting the research objectives. Now, in the final lines of the fourth paragraph of the introduction, we have added the following:**

***“However, despite the increasing use of AI in motor performance assessment, there is no comprehensive review examining its specific application in the assessment of BMS in preschool children, being a crucial stage for early detection and intervention. Moreover, the scope, limitations and validity of AI-based technologies in this context are not yet clearly systematized. Therefore, it is required to synthesize existing knowledge and guide the development of more accurate and accessible assessment tools”***

**Methods and discussion:**

 1. Lack of Detail in Study Selection Criteria

 While the article clearly defines the types of studies it targeted (engineering, substantive validation, and use of AI-related technologies), it doesn’t provide much detail on what specific inclusion and exclusion criteria were applied beyond these categories. For example, were studies excluded for reasons like sample size, study design, or methodological rigor?

 Suggestion: Provide more specific inclusion and exclusion criteria. Were only randomized controlled trials considered? Were there any restrictions based on the publication type (e.g., peer-reviewed articles only)?

**We have revised the manuscript and added more detailed inclusion and exclusion criteria. Specifically, in the third paragraph of methods, we added: *"We also defined the following criteria for the search: 1) studies in preschool-aged children (3 to 6 years), 2) studies in which the motor ability (motor or play skills) of the child was assessed using AI-related technologies for motion detection, and 3) studies in which at least one of the basic motor skills described in the literature (running, jumping, kicking, throwing, or catching a ball) was measured. In addition, we excluded 1) studies that did not clearly describe the AI-related technology used or developed, 2) opinion articles, editorials, or narrative reviews without empirical data and 3) gray literature (e.g. theses, dissertations, or non-peer-reviewed reports)."***

 2. Vague Explanation of Search Strategy

 The search strategy mentions specific databases but does not explain the rationale for selecting these particular sources over others. Are there other databases relevant to the field that might have been excluded? How were the keywords selected, and were any synonyms or related terms considered?

 Suggestion: Provide a clearer justification for the selection of these particular databases and search terms. Did you consider any grey literature (e.g., theses, dissertations, reports from non-peer-reviewed sources) to broaden the search?

**We've added clearer justification for our selection of databases and search terms. Specifically, we added in the methods section, subsection Search strategy, the following two paragraphs:**

**" *We searched for studies published before January 30, 2023 in the target publications in Medline (SCR_002185), Web of Science (SCR_022706), IEEE (SCR_008314), and EBSCO (SCR_022707). These databases were selected because they specialize in biomedical, engineering, and multidisciplinary research, ensuring that we captured relevant studies in health sciences, AI applications, and motion analysis.***

***Search terms included keyword combinations such as “child,” “preschool,” “basic motor skills,” “artificial intelligence,” “motion sensing,” and “calibration,” along with related terms and synonyms identified through a preliminary literature review (keywords) and controlled vocabulary (MeSH terms). The full search strategy and complete list of search terms are available here (42)."***

**Likewise, we cite the complete search strategy as number 42.**

  

1. Details on the Rayyan Platform

The mention of the Rayyan platform for managing studies is helpful, but the text doesn't clarify whether Rayyan was used for the full review process or just the initial screening. The review process appears to be manual in nature, but it could benefit from some details on how disagreements were handled between reviewers (e.g., was there a consensus meeting or did one reviewer have final say on the study’s inclusion?).

**Our team has detailed how Rayyan was used during the review process. Specifically, in the methods section, subsection Search strategy, in the third, fourth and fifth paragraph, we add:**

***"The search formulas were applied to the databases and all the files were exported in RIS format. Then, to ensure an objective selection process, these identified files were uploaded to the Rayyan platform which facilitated blind selection by the reviewers and expedited the identification of duplicates.***

***The selection process consisted of two phases. In the first phase, titles and abstracts were reviewed by two independent groups (each consisting of two previously trained medical students). To minimize selection bias, the Rayyan blinding function was used, which prevented reviewers from identifying the decisions of the other reviewers until the final selection phase. In addition, allocation of studies to reviewers was randomized within each group to further reduce potential bias. In case of disagreement, a consensus discussion was held among the reviewers. If consensus could not be reached, the principal investigator made the final inclusion decision.***

***In the second phase, a full-text review was performed following the same procedure, ensuring consistency and methodological rigor. The final set of studies was determined after resolving all discrepancies through consensus discussions and the intervention of the principal investigator."***

  

1. Potential Bias in Study Selection

The method states that the initial review of titles and abstracts was done by two independent groups, but there is no mention of any specific strategies to minimize bias during this phase. Given that the authors are likely familiar with the topic, it could be helpful to acknowledge how any biases in study selection (e.g., confirmation bias or publication bias) were minimized.

 Suggestion: Mention strategies to reduce bias in study selection. For example, were studies randomly assigned to reviewers, and was there a protocol to ensure that preconceived notions didn’t influence the selection process?

**Our team has added information about the process to minimize bias during study selection. Specifically, in the methods section, Search Strategy subsection, in the fourth and fifth paragraphs, we add:**

***"The selection process consisted of two phases. In the first phase, titles and abstracts were reviewed by two independent groups (each consisting of two previously trained medical students). To minimize selection bias, the Rayyan blinding function was used, which prevented reviewers from identifying the decisions of the other reviewers until the final selection phase. In addition, allocation of studies to reviewers was randomized within each group to further reduce potential bias. In case of disagreement, a consensus discussion was held among the reviewers. If consensus could not be reached, the principal investigator made the final inclusion decision.***

***In the second phase, a full-text review was performed following the same procedure, ensuring consistency and methodological rigor. The final set of studies was determined after resolving all discrepancies through consensus discussions and the intervention of the principal investigator."***

 5. Limited Details on Data Extraction Process

 While the data extraction form is outlined with categories such as general information, engineering, substantive validation, and use, there is little detail on how the data were extracted from the studies. For example, was any kind of reliability check conducted between reviewers, or were discrepancies resolved through discussion?

 Suggestion: Provide more details on the data extraction process, such as whether two independent reviewers performed the extraction and how discrepancies were resolved. Additionally, were any statistical methods used to assess inter-rater reliability in the extraction process?

**We have updated the paragraph by adding information in the methods section, Data extraction subsection. Now the text says the following:**

***"Data extraction was performed in a structured manner using a pre-designed*** ***form*** ***(43). To reduce errors and improve the accuracy of the extracted data, one peer reviewer performed the initial extraction and a second peer independently verified the information. Any discrepancies in the extraction were reviewed jointly and/or, with the intervention of the principal investigator. Cross-checks were implemented to ensure the consistency of the information collected."***

  

1. Lack of Clarity on Data Analysis

The description of data analysis mentions the use of descriptive statistics (frequencies and percentages) and the COSMIN standards for evaluating psychometric properties. However, there is no clear explanation of how the data were analyzed or how the results were synthesized. Were any meta-analysis or qualitative synthesis methods considered? How were the studies compared and summarized?

 Suggestion: Provide more clarity on how the data were synthesized. Was any quantitative or qualitative analysis beyond simple descriptive statistics performed, such as meta-analysis or thematic analysis? Did you perform a subgroup analysis for specific types of AI technologies or specific study characteristics?

**Since our study was a scoping review, we did not conduct a meta-analysis. Instead, we opted for a narrative synthesis of the results, presented in tables of frequencies and percentages. We also used the COSMIN standards, whose scoring and rating process is described in their original report, which we also reference in our study.**

**Specifically, our text, in the methods section, subsection "Data analysis," in its first paragraph, states the following: *"All data collected were summarized as categorical variables, organized and presented in tables, using descriptive statistics such as simple frequencies and percentages. Since this was a scoping review, a narrative synthesis was used to summarize the findings of the studies, focusing on the characteristics and psychometric properties evaluated according to COSMIN standards."***

  

1. Lack of Discussion on Potential Confounding Variables

There is a brief mention of the use of psychometric standards (COSMIN) to evaluate AI technologies, but the article doesn't discuss how confounding factors (e.g., differences in sample size, age groups, or types of technology used) might impact the validity of the conclusions. Were these factors taken into consideration in the evaluation process?

 Suggestion: Acknowledge potential confounding variables and how they might affect the validity of the studies reviewed. How did the authors control for these variables, if at all, in the synthesis process?

**We would like to clarify that, since our study is a *scoping review* focused on the mapping and characterization of AI-based technologies used to assess motor skills in children, we did not aim to establish causal or correlational relationships between variables. Therefore, the methodological approach does not involve controlling or statistically analyzing confounding variables, as would be expected in primary empirical studies. The purpose of this review was to identify and describe the technologies developed, their applications, and the reported psychometric dimensions.**

**Nonetheless, we have incorporated a critical reflection on the differences in sample sizes, age ranges (expressed in both years and months), and the types of technologies used, acknowledging these as limitations that could influence the validity and generalizability of the findings reported by the included studies.**

**Specifically, the last lines of the limitations section say the following: *"This review did not aim to analyze associations between variables; however, variability in sample sizes, age ranges, and types of AI-based technologies used across studies may affect the comparability and generalizability of the findings. These differences should be considered when interpreting the results and highlight the need for more standardized approaches in future research."***

 8. Unclear Rationale for Data Collection Methods

 In the section about data extraction, the mention of "feasibility and usability" as part of the data form is important but lacks further context. Was there a specific framework used to evaluate these factors? Were the usability and feasibility criteria standardized across the included studies?

 Suggestion: Provide more information on the frameworks or criteria used to assess usability and feasibility. Were these evaluations subjective or based on standardized measures? Clarifying this would help readers understand how the data extraction process assessed these aspects.

 Discussion   -    Discuss usability, cost-effectiveness, and accessibility challenges. -Emphasize the need for interdisciplinary collaboration in future validation studies. -Propose specific psychometric properties (e.g., construct validity) to prioritize  future AI validation research. - Add practical recommendations for improving AI tools (e.g., integrating psychometric standards, improving usability for non-specialists).

**Since no standardized framework was used to assess feasibility and usability, this is an aspect that limits the interpretation and comparison of the results among the included studies. We state this information in the limitations.**

**Specifically, we added to the beginning of the limitations:: *"Also, although this review was based on COSMIN standards to assess the psychometric quality of AI-related technologies, due to the heterogeneity observed in the included studies, no specific adjustments were made to control for possible confounding variables. Therefore, the conclusions need to be interpreted with caution. It is recommended that future research address these factors and use control methods to provide more generalizable conclusions. Furthermore, feasibility and usability were extracted only if the reviewed studies explicitly reported having done so in their analysis of AI-related technologies. Therefore, further studies should evaluate these analyzes using a standardized framework."***

**In response to comments on discussion, we have expanded this section on implications. Specifically, we added the following paragraphs in implications:**

***"To facilitate use, developers could conduct studies that evaluate the acceptance, ease of use, cost-effectiveness, and accessibility of these technologies. For example, most technologies rely on sensors and monitors that, while accurate, can be costly, require specialized training, and can be difficult to implement in real-world settings for physicians, teachers, therapists, or practitioners unfamiliar with these tools. In addition, disparities in access to advanced technologies may limit their adoption, particularly in low-resource settings.***

***Also, these types of technologies may be closer to more universal and cost-effective devices, such as video cameras, smartphones, and tablets, that can assess and report motor skills in real time. However, addressing these challenges requires a collaborative and interdisciplinary approach. Future validation studies should involve experts from multiple fields, including engineers, healthcare professionals, educators and policy makers, to ensure that these technologies are not only accurate, but also practical, scalable and accessible to diverse populations.***

***New validation studies of these technologies should include validation standards for BMS tests, prioritizing key psychometric properties such as construct validity, criterion validity, reliability, measurement error, among others. To make this possible, engineering teams could incorporate specialists in psychometrics, developmental therapy and medicine to work collaboratively. This multidisciplinary approach will facilitate the integration of medical knowledge and psychometric standards into future software releases, improving both measurement accuracy and practical usability. Finally, developers should consider providing open source code or detailed methodological documentation, which will allow for further refinement, replication, and clinical adaptation of these technologies in future research and real-world applications."***
